# Quality of life and therapeutic regimen management in onychomycosis patients and in vitro study of antiseptic solutions

**DOI:** 10.1038/s41598-021-92111-4

**Published:** 2021-06-17

**Authors:** Vasco Silva-Neves, Vitor Hugo, Paulo Alves, João Costa Amado, Carla Pais-Vieira, Fátima Sousa, Fátima Cerqueira, Eugénia Pinto, Miguel Pais-Vieira

**Affiliations:** 1 Centro de Investigação Interdisciplinar em Saúde, Instituto Ciências da Saúde, Universidade Católica Portuguesa, Rua de Diogo Botelho, 1327, 4169-005 Porto, Portugal; 2 Hospital das Forças Armadas-Polo Porto, Avenida da Boavista, 4050-113 Porto, Portugal; 3 Centro Hospitalar Universitário São João, Alameda Prof. Hernâni Monteiro, 4200-319 Porto, Portugal; 4 FP-ENAS/CEBIMED, Fernando Pessoa Energy, Environment and Health Research Unit/Biomedical Research Center University Fernando Pessoa, Praça 9 de Abril, 349, 4249-004 Porto, Portugal; 5 Health Sciences Faculty, University Fernando Pessoa, Rua Carlos da Maia, 296, 4200-150 Porto, Portugal; 6 Interdisciplinary Centre of Marine and Environmental Research (CIIMAR/CIMAR), University of Porto, Terminal de Cruzeiros do Porto de Leixões, Av. General Norton de Matos s/n, 4450-208 Matosinhos, Portugal; 7 Laboratory of Microbiology, Biological Sciences Department, Faculty of Pharmacy of University of Porto, Rua Jorge Viterbo Ferreira nº 228, 4050-313 Porto, Portugal; 8 iBiMed-Institute of Biomedicine, Department of Medical Sciences, University of Aveiro, Campus Universitário de Santiago, Agra do Crasto-Edifício 30, 3810-193 Aveiro, Portugal

**Keywords:** Fungi, Epidemiology, Fungal infection, Disease prevention, Public health, Quality of life, Therapeutics

## Abstract

Onychomycosis or tinea unguium (EE12.1) and Onychomycosis due to non-dermatophyte moulds (1F2D.5) (OM) is a fungal infection of the nail plates with a high prevalence that often affects vulnerable people with co-existing health problems. Gold standard pharmacological treatments for onychomycosis have been associated with low success rates and increasing antifungal resistance, suggesting that treatment outcome is dependent on multiple variables. Here, the prevalence of OM and quality of life were characterized in two vulnerable populations—Hospital patients and Homeless people. Comparing both groups, the most prevalent fungal species were identified in Hospital patients. Then, the in vitro fungicidal properties of the antiseptics povidone-iodine, polyhexamethylene biguanide-betaine, octenidine dihydrochloride, and a super-oxidized solution against two ATCC strains (*Candida albicans* and *Aspergillus niger*) and three clinical fungal isolates from Hospital patients (*Candida parapsilosis*, *Trichophyton interdigitale*, and *Trichophyton rubrum*) were tested. OM prevalence was high in both patient groups studied, who also reported a reduction in quality of life and concerns about the state of their feet. In addition, Hospital patients had a non-negligent therapeutic regimen management style. Antiseptics tested in vitro revealed antifungal properties. As antiseptics are low-cost and easy to apply and have few iatrogenic effects, the demonstration of fungicidal properties of these solutions suggests that they may constitute potential supportive therapeutics for OM.

## Introduction

Onychomycosis (OM) is a fungal infection of the nails plates that is often caused by dermatophytes and is characterized by nail plate thickening and dystrophy. OM is estimated to infect 8.9% (95% CI 4.3–13.6) of the population in Europe^1^. Epidemiological studies have indicated that the OM prevalence tends to be higher in older people and vulnerable populations^2^. Some studies have revealed prevalence rates of 46% in diabetic patients with nail plate problems^3^ and 51.3% in those that do not practice daily hygiene targeting the feet^4^. In addition, patients with peripheral vascular disease are at increased risk of onychomycosis^5^. The dermatophyte most frequently identified in OM is *Trichophyton rubrum* followed by *Trichophyton mentagrophytes*^6,7^. However, several studies have also reported OM with non-dermatophyte moulds^8,9^.

Currently, several treatments for OM are used, namely, laser treatment, surgery, debridement, and pharmacological interventions^10–12^. The gold standard pharmacological treatment for OM is a continuous or intermittent therapeutic scheme including amorolfine, ciclopirox, terbinafine, and itraconazole^2,6,13^. Despite the multiple approaches and treatments available, the success rate remains relatively low^13^, with infection recurrence rates ranging from 40–70%^13^. Recent studies have suggested that this low success rate and the recurrence of infection may be, in part, explained by the iatrogenic effects of medication, long duration the treatment, associated cost, and self-care commitment and style^13^. These studies have also suggested that approaches with fewer iatrogenic effects, which can reduce treatment length and expenses or promote self-care, may have the potential to improve current treatments and recurrence success rates. One group of substances with several of these characteristics are antiseptic solutions. These are inexpensive, generally well tolerated, and can be easily applied at home without significant associated health risks. However, there is relatively scarce evidence on their efficacy in OM^14^. To improve our current knowledge on the potential of antiseptic solutions as supportive therapies for OM treatment, four common antiseptics were tested against the dermatophyte, non-dermatophyte, and yeasts causative agents of OM. Testing this hypothesis requires a determination of which fungal species and antiseptic solutions should be studied while accounting for complex variables that have been previously demonstrated to influence the outcome of other treatments, namely, quality of life (QOL) and therapeutic regimen management style (TRMS).

To achieve this goal, we performed a three-part study to (i) characterize the prevalence of OM among patients from two groups (Hospital patients and Homeless people) and identify the most prevalent fungal species in Hospital patients; (ii) characterize self-care and QOL in both groups and TRMS in Hospital patients; and (iii) evaluate in vitro the fungicidal properties of antiseptics often used in clinical practice for infected wounds: povidone-iodine (PVP-I), polyhexamethylene biguanide-betaine (PHMB-B), octenidine dihydrochloride (OCT-D), and a super-oxidized solution (SOS) against two ATCC strains (*Candida albicans* and *Aspergillus niger*) and three clinical isolates from Hospital patients (*Candida parapsilosis*, *Trichophyton interdigitale* and *Trichophyton rubrum*).

## Methods

### Sampling, OM prevalence and identification of the etiological agents

A general Hospital population (Hospital Patients Group—HosPG) and a Homeless population (Homeless People Group—HomPG) were studied. The STrengthening the Reporting of OBservational studies in Epidemiology (STROBE) checklist was used to report the results^15^. All methods were performed in accordance with the relevant guidelines and regulations. Statistical analysis was performed using IBM SPSS Statistics for Macintosh, Version 26.0. Armonk, NY: IBM Corp. (https://www.ibm.com/analytics/spss-statistics-software).

**Figure 1:**
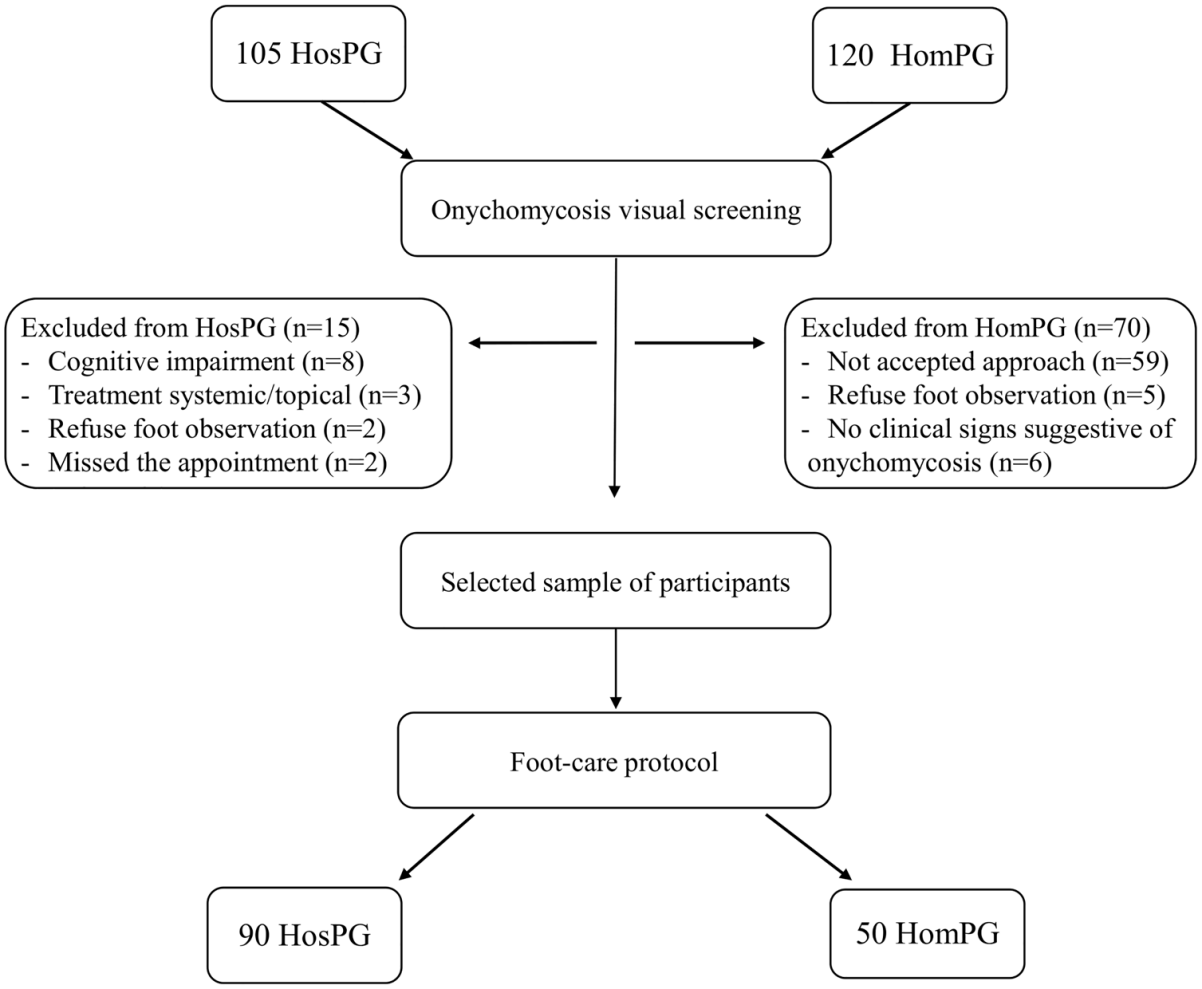
Hospital Patients Group (HosPG) and Homeless People Group (HomPG) flow diagram.

The screening protocol for the HosPG and HomPG is presented in Fig. 1.

#### Hospital patients group

A descriptive, cross-sectional study was performed between September 2018 and December 2019 to evaluate the prevalence of OM in the HosPG. A nonprobability convenience sample comprising ninety participants (Fig. 1) was enrolled. This observational study started with the initial referral of patients from specific departments (dermatology, endocrinology, podology, and internal medicine) who presented clinical signs of OM (nail plate thickening, yellowing, and dystrophy). Participants were contacted subsequently referral from these departments, after initial screening by a Physician, a Dermatologist, a Nurse expert, or a Podiatrist. The inclusion criteria were as follows: 18 years of age or older, no obvious cognitive impairment, and clinical signs suggestive of OM. Sociodemographic characteristics, clinical history, and signs and symptoms related to OM were annotated and later analysed for each patient.

In all patients, feet were initially washed with mild soap and water, followed by antisepsis of the skin and nail plates with an alcoholic solution (70% v/v). Nail plates were then scraped with a sterile scalpel blade, and collected in a sterile container and sent to the laboratory. All samples were analyzed by direct microscopy and cultivable methods. Direct examination was performed based on microscopic preparations with 10–20% KOH and fungal cultivation on Sabouraud gentamicin chloramphenicol 2 (SGC2) and Sabouraud agar with chloramphenicol and cycloheximide. From positive culture, the present fungi were isolated and identified (in each case, pure cultures were obtained finally). Filamentous fungi, such as dermatophytes, were identified by macroscopic and microscopic observations, and yeasts were identified by a VITEK^®^ 2 Compact platform.

Diagnosis of dermatomycoses was based on the detection of septate hyphae by direct microscopic examination and cultivable methods with subcultures that were validated by two different pathologists. These analyses consisted of observation of morphology (pigmentation of the surface and reverse sides, topography, texture and rate of growth) and microscopic characteristics (size and shape of macroconidia and microconidia, spirals, nodular organs) in combination with biochemical and physiological tests (nutritional requirements, temperature tolerance, enzyme production). When an accurate identification could not be made using these methods, the samples were sent to a collaborating university hospital (Centro Hospitalar Universitário do Porto, Portugal) to be confirmed with an additional combination of methods, namely: urea broth, bromocresol purple agar, trichophyton agar. In addition, matrix-assisted laser desorption ionization-time of flight mass spectrometry (MALDI-TOF MS) was used to confirm the accurate identification of the isolates.

#### Homeless people group

A descriptive, cross-sectional study was performed between October 2018 and May 2019 to define the prevalence of OM in the HomPG. The study population consisted of a nonprobability convenience sample comprising fifty participants (Fig. 1). In the present research, we chose a convenience sample from an institution attended by Homeless people because it was essential to approach people without changing their quotidian routine. The study was conducted during lunch time (i.e., when Homeless people went to the institution for a free meal). One researcher contacted and kept track of participants who met the inclusion criteria (same as those for the HosPG). Those who were selected to join the study were provided a detailed explanation, and signed informed consent was provided. For those who agreed to participate, health care and comfortable foot care were provided. Foot care consisted of washing the feet with a soap solution, followed by cleaning and disinfection with alcoholic solution. Then, a nail plate sample was collected, and a dermatophyte test (Diafactory Tinea Unguium, Japan) based on immunochromatography was immediately performed. This is a quick method that uses mouse monoclonal antibodies on a nitrocellulose membrane and which has a high sensitivity for dermatophyte fungi (98.0%) and limited sensitivity for non-dermatophyte fungi (specificity, 88.2%). Nail plate samples were collected to a test tube and allowed to react for a period of 5 min. The final colour present in the test tube was then compared with the test strip. During this contact time, notes were taken on the characteristics of acute and chronic injuries, and participants’ biographic and health data were collected.

### QOL and therapeutic regimen compliance

In part two of the study, the impact of OM on QOL was evaluated in a group of HosPG and HomPG patients. In the HosPG, therapeutic regimen compliance was evaluated using the TRMS questionnaire with a grounded theory approach (explanatory theory on self-management in chronic illness)^16^. This self-report is composed of two parts. The first uses a scale ranging from 0—totally disagree to 4—totally agree and allows the identification of personality traits and attitudes towards the disease and the therapeutic regimen. The second part contains a scale ranging from 0—never to 4—always to evaluate the subjects’ perceived behaviour towards the therapeutic regimen.

### Evaluation of fungicidal potential of antiseptic products

Fungicidal activity was evaluated according to the European Standard EN 1275 “Chemical disinfectants and antiseptics—“*Quantitative suspension test for the evaluation of basic fungicidal or basic yeasticidal activity of chemical disinfectants and antiseptics*”^17^. This standard allows the testing of the fungicidal activity of antiseptic products when diluted with water and a neutralizing formula. The common commercial antiseptic solutions PVP-I, PHMB-B, OCT-D, and SOS were tested at the highest concentration of 80%, diluted with water and added to a test fungal suspension. The obligatory test conditions required maintaining the mixture at 20 ± 1 °C under 15 min ± 10 s. Furthermore, this standard required the reference strains *C. albicans* ATCC 10231 and *A. niger* ATCC 16404 to evaluate fungicidal activity. In addition to testing under the standard conditions using the mandatory microorganisms indicated by EN 1275, the fungicidal activity was also evaluated against the clinical isolates *C. parapsilosis* (FF179), *T. interdigitale* (FF164), and *T. rubrum* (FF165), which were the most prevalent fungal species isolated from the HosPG.

The SOS was tested as 95% solution due to the quick loss of properties when the solution was open. The neutralizer used was D/E neutralizing broth (NCM0047), which offered stability for all the antiseptics.

The numbers of CFUs (colony forming units) after treatment under test conditions were counted, and the log reduction was calculated. Following the EN 1275 recommendations, only when the reduction in CFUs was equal to or higher than log 4 was fungicidal activity considered.

Statistical analysis was performed using the Statistical Package for the Social Sciences (SPSS), version 26.0. The data are expressed as frequencies and percentages. Results were considered significant at an alpha value of 5%. The log reduction was calculated according to the formula described in EN 1275^17^, i.e., as the log reduction difference between the number of CFU/ml test suspensions and the number of CFU/ml experimental suspensions.

### Ethics approval

This study was approved by the Ethics Committee of the Universidade Católica Portuguesa, Centro de Investigação Interdisciplinar em Saúde [CE.C. (11)2018)] and the Ethics Committee of Armed Forces Hospital (SEFT-Estudos-0002/2017-1). Informed consent was signed by all the participants after careful explanation of the study. Subjects participated in the study at no cost. Confidentiality and privacy of participants' personal data was guaranteed at all times according to the European Union General Data Protection Regulation 2016/679.

## Results

**Figure 2:**
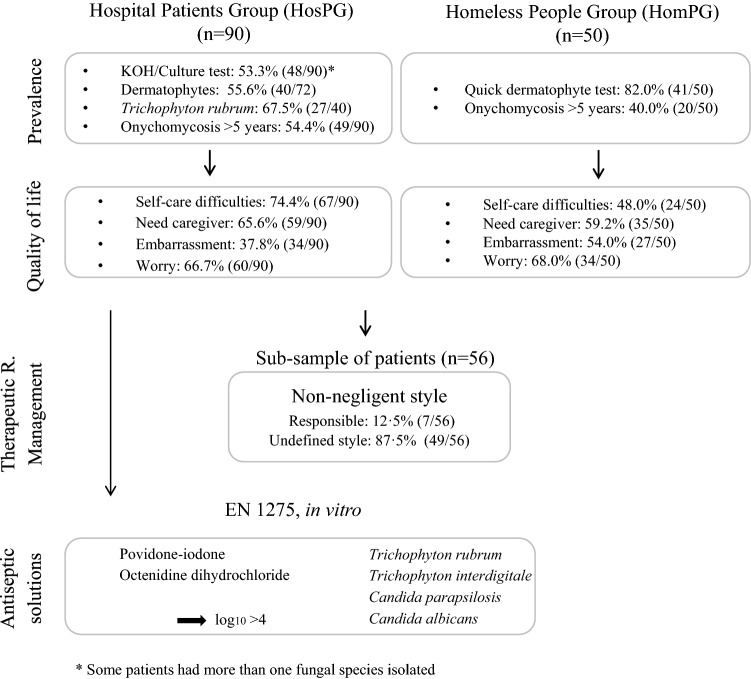
Comparison of results from the Hospital Patients Group (HosPG) and Homeless People Group (HomPG).

As the present study comprised multiple parts, the results will be presented as follows: (i) comparison of demographic and clinical history data of the HosPG and a HomPG; (ii) OM classification for both groups studied; (iii) description of QOL and TRMS in the HosPG; (iv) comparison between results obtained by direct examination in 10–20% KOH and based on cultivable methods in the HosPG and the results of quick dermatophyte tests in the HomPG; (v), comparisons of in vitro antifungal properties of antiseptics against three strains of the most prevalent yeast and dermatophyte species isolated from the HosPG (the summary of all the results are presented in Fig. 2.

### Demographics of the hospital patients group and homeless people group

Table 1 shows the demographic and clinical data obtained in the prevalence study of the HosPG and HomPG. A total of 105 patients from the general Hospital population were screened, and 85.7% (n = 90) presented clinical signs of OM. Moreover, among the Homeless population, a total of 120 people were screened, of which 41.7% (n = 50) were enrolled and constituted the HomPG.

**Table 1:** Demographic and clinical data of the Homeless People Group (HomPG) and Hospital Patients Group (HosPG).

	HomPG	HosPG
**Age group at registration (years)**	n = 50	n = 90
Mean (SD)	53 (11.1)	65 (15.4)
≥ 18 ≤ 40	3 (6%)	7 (8%)
> 40 ≤ 60	34 (68%)	22 (24%)
> 60	13 (26%)	61 (68%)
**Sex**	n = 50	n = 90
Female	8 (16%)	32 (36%)
Male	42 (84%)	58 (64%)
Education		
Basic education first cycle	29 (58%)	38 (42%)
Basic education second and third cycle	13 (26%)	17 (19%)
Secondary education	5 (10%)	25 (28%)
Higher education	3 (6%)	10 (11%)
**Clinical history (number of factors)**	n = 113	n = 165
Oncological disease	1 (1%)	13 (8%)
Visual or hearing disability	5 (4%)	5 (3%)
Psychiatric disorder	5 (3%)	3 (2%)
Accidents/trauma	7 (6%)	2 (1%)
Surgical history	7 (6%)	30 (18%)
Chronic disease	11 (10%)	28 (17%)
Cardio and cerebrovascular disease	13 (12%)	58 (35%)
Commitment to mobility and self-care	13 (12%)	10 (6%)
Infectious disease	21 (19%)	12 (7%)
Addiction (alcohol, tobacco, drugs)	30 (27%)	3 (2%)
**Signs and symptoms of onychomycosis**	n = 50	n = 90
Pain	30 (60%)	32 (36%)
Soreness	37 (74%)	59 (66%)
Injury	40 (80%)	27 (30%)
Thickening	46 (92%)	81 (90%)
Yellowing/discolouration	50 (100%)	90 (100%)
**Impact on quality of life**	n = 50	n = 90
Daily hygiene for feet	14 (28%)	66 (73%)
Caregiver needed	15 (30%)	59 (66%)
Self-care difficulties	24 (48%)	67 (74%)
Use changing rooms or swimming pools	25 (50%)	9 (10%)
Shame/embarrassment	27 (54%)	34 (38%)
Worry	34 (68%)	60 (67%)
Wearing inadequate footwear	50 (100%)	8 (9%)
**Onychomycosis prevalence**	n = 50	n = 90
Positive dermatophytes test	41 (82%)	–
Laboratory diagnosis (KOH + culture)	–	48 (53%)

Data are n (%). *HomPG *Hospital Patients Group, *HosPG *Homeless People Group.

### Onychomycosis classification

The most prevalent pattern in the HosPG was the *mixed pattern* (21.4%; 191/894 toenails observed), followed by *Distal and Lateral Subungual Onychomycosis *(*DLSO*) (20.6%, 184/894). *Total Dystrophy Onychomycosis *(*TDO*) (2.7%; 24/894), *Superficial White Onychomycosis* (1.0%; 9/894), and the *Proximal Subungual Onychomycosis* (0.7%; 6/894) were less prevalent. Slightly more than half of the toenails (54.4%, 486/894) presented no alterations.

In the HomPG, the main pattern was *DLSO* (49.9%; 249/499 toenails observed), followed by the *Mixed Pattern Onychomycosis* (12.8%, 64/499) and *TDO* (3.8%, 19/499). One-third of the nails plates (33.1%; 165/499) presented no alterations. The *DLSO* pattern was observed in all nails plates, particularly in the first toenail (hallux) (68.0%, 34/50 right foot; 64.0%, 32/50 left foot). The 5th toenail presented the most heterogeneous patterns. These patterns occurred symmetrically in both feet, with minimal differences between the right and left foot. In addition, many participants (68.0%; 34/50) had between three and five nails plates affected on each foot. No differences were found between the right and left foot (*p* = 0.59).

### QOL in patients suffering of onychomycosis

Analysis of the QOL indicated that for 73.3% (66/90) of the subjects in the HosPG, foot hygiene was part of their daily routine. Close to 65% reported needing help from a caregiver to perform daily hygiene activities (59/90), and 74.4% reported having self-care difficulties (67/90). Shame or embarrassment was reported by 37.8% (34/90), with 66.7% (60/90) reporting being worried about the condition of their feet.

In the HomPG, foot hygiene was not part of their daily routine (72.0%; 36/50), with 70.0% (35/50) indicating that they needed help from a caregiver to perform daily hygiene activities. Slightly less than half of the subjects (48.0%, 24/50) reported having self-care difficulties. Furthermore, 54.0% (27/50) reported shame or embarrassment, and 68.0% (34/50) reported being worried about the condition of their feet.

When questioned about the duration of their OM, the majority of the respondents in the HosPG reported that their OM had been present for longer than 5 years (54.4%, 49/90), while 8.9% (8/90) did not know or did not answer. In the HomPG, 28.0% (14/50) did not know or did not answer the question regarding foot hygiene frequency and reported that their OM had been present for longer than 5 years.

These results indicated that the prevalence of OM was high and had been present for several years in both groups of patients. Subjects had self-care difficulties and were worried about the condition of their feet; therefore, their QOL was compromised.

### Non-negligent therapeutic regimen management

**Figure 3:**
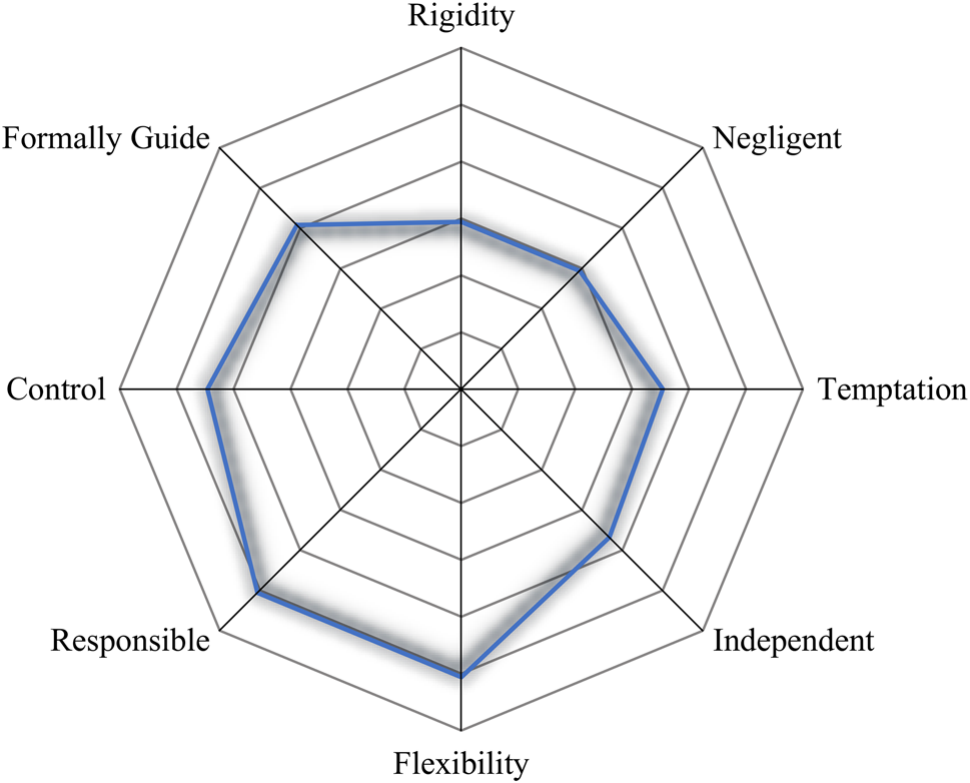
Chart of the TRMSs of patients with OM.

To gain further knowledge about the possible causes of the low success rate of current OM treatments, the Questionnaire on Personality Traits and Attitudes Towards the Disease and the Therapeutic Regimen^14^ was administered to 62.2% (56/90) of the HosPG. The majority of participants presented an *undefined style* (85.7%, 48/56), and 14.3% (8/56) were classified as *responsible* (i.e., involving the constructs of control and flexibility). Even though a clear style could not be defined for the majority of the subjects, the characteristics of *rigidity* and *temptation*, which are more often associated with the *negligent* type, were largely absent in this subsample. This multivariable deviation from the negligent characteristics can be observed in the radar chart presented in Fig. 3. These results indicated that the sub-sample analysed was mostly characterized by a non-negligent regimen management style.

### Detection of fungi in clinical materials and identification of isolates to the species level

A total of 90 HosPG samples were collected and analysed by direct microscopy and cultivable methods; a positive result of direct microscopical examination of nail plate samples was present in 52.2% (47/90) and 53.3% (48/90) in case of founded fungal cultures. Among the 72 fungal isolates obtained by mycological cultures on Sabouraud agar 55.6% (40/72) were dermatophytes. *T. rubrum* was the most common fungal species isolated from these samples, at 37.5% (27/72 isolates), and the most frequently isolated dermatophyte, at 27/40 (67.5%) (Table 2).

**Table 2:** Distribution of species identified in nail plate samples of Hospital Patients Group (HosPG).

	n	%
**Dermatophytes**		
*Trichophyton rubrum*	27	37.5
*Trichophyton interdigitale*	11	15.3
*Epidermophyton floccosum*	1	1.4
*Trichophyton violaceum*	1	1.4
Subtotal	40	55.6
Nondermatophytes*		
*Aspergillus* sp.	5	6.9
*Penicillium* sp.	5	6.9
*Chaetomium* sp.	4	5.6
*Fusarium* sp.	2	2.8
*Alternaria* sp.	2	2.8
*Aspergillus niger*	2	2.8
*Acremonium* sp.	1	1.4
*Aspergillus fumigatus*	1	1.4
*Aspergillus penicillioides*	1	1.4
*Cladosporium* sp.	1	1.4
Subtotal	24	33.3
Yeasts*		
*Candida parapsilosis*	5	6.9
*Candida albicans*	1	1.4
*Candida guilliermondii*	1	1.4
*Trichosporon mucoides*	1	1.4
Subtotal	8	11.1
Total	72	100

Data are n (%).

*Some strains are colonization or contaminants.

The diagnosis of OM in the HomPG with a kit dermatophytes test revealed that out of the fifty participants with clinical signs of tinea unguium*,* 82.0% (41/50) had a positive test result.

### In vitro antifungal activity of antiseptics

As OM had a high prevalence and was associated with a reduction in QOL (in the both studied groups of patients) and with a non-negligent therapeutic regimen style (in the HosPG), whether antiseptic solutions have potential as a simple and safe co-adjuvant treatment for OM was considered. For this, the in vitro effects of different antiseptic solutions were tested against the ATCC strains and the three most prevalent fungal species identified in the group of HosPS patients.

European Standard EN 1275 was followed for testing commercial formulas of PVP-I, PHMB-B, OCT-D, and SOS. The results obtained after log_10_ reduction (logR) are presented in Table 3.

**Table 3:** Results of antiseptic solution testing for antifungal activity, presented in log reduction values.

	PVP-I	PHMB-B	OCT-D	SOS
*Aspergillus niger*	3.10 ± 0.10 (n = 3)	3.10 ± 0.10 (n = 3)	3.10 ± 0.10 (n = 3)	3.10 ± 0.10 (n = 3)
*Candida albicans*	4.39 ± 0.12 (n = 2)	4.39 ± 0.12 (n = 2)	4.39 ± 0.12 (n = 2)	3.44 ± 0.75 (n = 3)
*Candida parapsilosis*	4.50 ± 0.49 (n = 4)	4.63 ± 0.08 (n = 2)	4.63 ± 0.08 (n = 2)	3.54 ± 0.56 (n = 5)^a^
				4.35 ± 0.72 (n = 3)^b^
*Trichophyton interdigitale*	4.29 ± 0.39 (n = 2)	4.40 ± 0.34 (n = 3)	4.29 ± 0.39 (n = 2)	3.62 ± 0.68 (n = 4)
*Trichophyton rubrum*	4.18 ± 0.01 (n = 2)	3.70 ± 0.53 (n = 6)	4.18 ± 0.36 (n = 4)	3.23 ± 0.23 (n = 8)

Values of log_10_ reduction mean and standard deviation. In parentheses; *n *number of valid tests. *PVP-I *povidone-iodine, *PHMB-B *polyhexamethylene biguanide-betaine, *OCT-D *octenidine dihydrochloride, *SOS *super-oxidized solution. ^a^Tested compound at 80% (according to the protocol); ^b^Tested compound at 95%.

Considering the reference strains of fungi used for the test validation, for *A. niger,* we verified a log_10_ reduction in antiseptic activity less than 4 (logR = 3.10 ± 0.10), indicating that no fungicidal activity for the four antiseptics could be detected according to EN 1275. However, for *C. albicans,* fungicidal activity was found for PVP-I, PHMB-B, and OCT (logR = 4.39 ± 0.12). For this species, the SOS did not produce consistent results, with the three valid tests indicating a mean log_10_ reduction of 3.44 ± 0.75.

Testing of the clinical strains revealed fungicidal activity against *C. parapsilosis* in three of the four antiseptics: PVP-I (logR = 4.50 ± 0.49), and PHMB-B and OCT-D (the same and consistent results of log_10_ reduction of 4.63 ± 0.08) In contrast, the SOS at the same concentration of 80% showed a logR less than 4 (logR = 3.54 ± 0.56), similar to that observed for *C. albicans*, with no fungicidal activity. However, when we tested the SOS at 95% (optional condition according to the protocol), a logR of 4.35 ± 0.72 was determined for *C. parapsilosis*.

For the dermatophytes, the antiseptics PVP-I and OCT-D induced similar log_10_ reductions for *T. interdigitale*, at logR = 4.29 ± 0.39, and *T. rubrum* induced log_10_ reductions of logR = 4.18 ± 0.01 and logR = 4.18 ± 0.36, respectively; fungicidal activity was observed for both antiseptics. The PHMB-B solution presented different antifungal potency depending on the dermatophyte species, with *T. interdigitale* being more susceptible than *T. rubrum*. In fact, fungicidal activity was observed against *T. interdigitale,* at logR = 4.40 ± 0.34, while for *T. rubrum,* no fungicidal effect was observed (logR < 4; 3.70 ± 0.53). Finally, the antiseptic SOS activity against dermatophytes was variable, with no fungicidal activity; the logR values ranged between 3.62 ± 0.68 for *T. interdigitale* and 3.23 ± 0.23 for *T. rubrum*.

## Discussion

In the present study, OM prevalence, QOL, and the therapeutic regimen were studied, and the in vitro antifungal properties of antiseptics were evaluated in the most prevalent clinical species from the HosPG. Approximately half (53.3%) of the HosPG tested positive for OM, with *T. rubrum* being the most prevalent fungus, as found in previous studies^1,2,13,18,19^.

The subjects reported self-care difficulties, need for a caregiver, and embarrassment as well as concerns about their feet. While no clear TRMS could be identified in most cases, the subjects presented characteristics associated with non-negligent management types. In the HomPG, OM was present in most of the subjects (82%). Several factors could account for the differences in the presence of dermatophyte fungi between the two samples in this study (40 dermatophyte positive/90; 44% in the HosSP and 41 dermatophyte positive/50; 82% in the HomPG). First, different tests were used to detect dermatophytes. The tests used in the HosPG (KOH + culture) (sensitivity 61%/56%, specificity 99%/99%, predictive of positive value 96.3%/99.4%, predictive of negative value 55.9%/52%, respectively)^20^ have greater specificity but worse sensitivity than the immunochromatography test used in the HomPG, even though it does not allow the identification of dermatophytes (sensitivity 98%, specificity 78%, predictive of positive value 84.8%, predictive of negative value 97%, agreement rate 89.1%)^21^. It should be noted that the test used for the HomPG was specific for dermatophytes. Therefore, it is possible that other fungi may have been present in the samples analyzed but were not detected. Second, in the HosPG, we considered the conjugated positive data between the two methods (KOH + culture), while in the HomPG, who received a rapid test, all positive results (from weak to strong positives) were considered. Third, as foot hygiene conditions in the HomPG were worse than those in the HosPG, higher prevalence results were expected.

The limitations of our study include a small, number of participants which were included in the study, particularly in the HomPG and the use of different diagnostic tests, and the absence of molecular methods in diagnosis. For the HosPG, the tests were able to be performed at the hospital’s laboratory; however, for the HomPG, a private laboratory would be necessary to perform the same tests. To avoid this, a quick diagnostic test was carried out on location, which was more easily accepted by Homeless people. In addition, the different ages of the populations and the homelessness condition may have affected the results. Also, only a limited number of strains were analyzed (representing the ones that were more prevalent in our samples) and therefore additional studies are required to determine if the susceptibility tests and conclusions are extensive to other species and strains. Last, it should also be considered that the diagnostic tests evaluated with culture and KOH were valid but are known to be inferior to PAS staining^20^. This means that the prevalence rate reported here may be underestimated.

An in vitro antimicrobial study revealed that povidone-iodine and octenidine dihydrochloride presented fungicidal activity against clinical strains of *T. rubrum* and *T. interdigitale,* as well against *C. parapsilosis* and *C. albicans.* Considering that the most common species isolated from OM are dermatophytes^6,10^, and in the present study, they were *T. rubrum* and *T. interdigitale*, as well as the yeasts *C. albicans* and *C. parapsilosis*, it can be concluded that PVP-I and OCT-D have broad spectrums to treat or prevent OM due to the most common fungal agents. Fungi in the genus *Aspergillus* may also be involved in OM, although this is rare^19^. According to the standard used, none of the studied antiseptics had fungicidal activity against *A. niger*.

The antiseptic solution PVP-I has already demonstrated efficacy in the skin and mucous membranes against viruses and bacteria, with microbial inactivation greater than 99.99%^22–24^. Additionally, the OCT-D solution has demonstrated good efficacy against skin microflora in multiple studies^22,24,25^, with stronger activity against *Staphylococcus epidermidis* and weaker activity against *Escherichia coli* and *C. albicans*^22^. Furthermore, this solution seems to represent a well-tolerated, safe and efficacious therapeutic choice for the treatment of *Pseudomonas* nail plate infection^26^. In our study, we verified identical reductions in fungal clinical strains by both antiseptic solutions.

PHMB has demonstrated its effectiveness against several microorganisms^11,27,28^*.* In textile tests, PHMB demonstrated good efficacy against *T. rubrum*, and the SOS demonstrated good efficacy against *T. mentagrophytes*^29^*.* However, a different result was observed in our study; PHMB had fungicidal activity (logR > 4) against *T. interdigitale* but not against *T. rubrum* (logR < 4). For *T. rubrum*, although a decrease in CFUs was observed, no fungicidal activity was found according to the standard test. While Hammer et al. (2011) reported activity of the SOS against *T. rubrum*^29^ using antimicrobial textiles, for our standard test, no fungicidal activity was observed toward dermatophytes and yeasts cells. Nevertheless, for both antiseptic solutions, it should be noted that we were comparing different tests. In the present study, we observed that the SOS activity decreased over time, suggesting a loss of activity after opening of the packaging. This was also identified in studies by Rossi-Fedele et al., who reported that stability was affected by storage conditions and exposure to light^30^. Additional testing with a new sample of SOS revealed stronger activity, and when we tested the older sample at 95%, we observed an increase in activity. However, this consideration could limit the use of this solution if it remains open for long periods.

OM has a worldwide impact due to its chronicity, with high resistance to treatments and frequent recurrence^2,31,32^. OM reduces QOL, triggering physical, mental and social problems affecting daily activities^33^. In patients with risk factors, it can cause severe cases of infection, with the need for differentiated health care, overloading health care services and increasing the cost of care^4,34^*.* Our results indicated that OM reduced the quality of life of patients; they also reported that they were concerned about the state of their feet and that they had non-negligent therapeutic regimen management styles. Therefore, our findings suggest that the potential of antiseptics in OM (e.g., prophylaxis, support therapy, or alone) should be further studied in vivo and in other strains.

Finally, a somewhat similar therapeutic has been proposed in a recent patent application involving a petrolatum-based solution to treat OM^35^.

## Conclusion

The present study indicates that OM affected the QOL of two different vulnerable populations and that these subjects reported being embarrassed and concerned about their feet. The TRMS of the Hospital patient sample revealed characteristics related to non-negligent management styles. In vitro antimicrobial evaluations of antiseptics revealed antifungal properties toward cells of the clinical fungi. These results support the need for further studies on the use of antiseptics in the treatment of OM.
